# Spontaneous rupture of bicornuate uterus in second trimester: A case report

**DOI:** 10.1016/j.ijscr.2025.111862

**Published:** 2025-08-24

**Authors:** Sagar Bishowkarma, Sasmit Ghimire, Raman Kumar Gurmaita, Manisha Gurung, Manvi Mukherjee, Hema Rijal

**Affiliations:** a Maharajgunj Medical Campus, Institute of Medicine, Tribhuvan University, Kathmandu, Nepal; b Department of Gynecology, Tribhuvan University Teaching Hospital, Kathmandu, Nepal

**Keywords:** Bicornuate uterus, Uterine rupture, Second trimester, Hemoperitoneum

## Abstract

**Introduction and importance:**

The bicornuate uterus is a rare congenital anomaly of the uterus. Complete uterine rupture in the first or second trimester associated with a bicornuate uterus is an infrequent and life-threatening presentation. Due to its rarity, the diagnosis is often overlooked, leading to potentially fatal outcomes.

**Case presentation:**

Here, we report a rare case of spontaneous complete rupture of the right horn of a bicornuate uterus in a 32-year-old woman at 20 weeks of gestation, who presented with abdominal pain and vomiting. On evaluation, rebound tenderness and guarding were present mimicking conditions such as acute appendicitis or ruptured ectopic pregnancy. On ultrasonographic evaluation, hemoperitoneum was suspected and a diagnosis of complete uterine rupture was made intraoperatively and managed by excision of the gravid rudimentary horn.

**Clinical discussion:**

At an early gestation, uterine rupture usually presents with a nonspecific clinical presentation; making its diagnosis more difficult. However, not all cases present these features; thus, it is essential to maintain a high degree of suspicion. This case highlights the importance of early recognition of uterine anomalies like bicornuate uterus to prevent life-threatening complications such as uterine rupture.

**Conclusion:**

Uterine rupture should be considered in pregnant women presenting with acute abdominal pain, hypovolemia, hemoperitonium, even in early pregnancy. Prompt surgical intervention is crucial to prevent maternal mortality.

The objective of this case report is to raise clinical awareness about the possibility of spontaneous uterine rupture in early pregnancy, particularly in women with undiagnosed uterine anomalies.

## Highlights

• Spontaneous rupture of a bicornuate uterus in the second trimester of pregnancy is very rare.
• Early diagnosis of uterine anomalies is crucial for preventing maternal and fetal morbidity.
• This case highlights the importance of timely surgical management in cases of uterine rupture.
• Diagnosis and management of a bicornuate uterus in pregnancy is challenging.
• This case guides clinicians in managing high-risk pregnancies effectively.

## 1 Introduction

The incidence of uterine anomalies ranges from 0.06 % to 38 % in the general population. Among various anomalies, a bicornuate uterus is a common type of uterine anomaly [1]. A bicornuate uterus is a congenital anomaly of the uterus that occurs when two Mullerian ducts fail to fuse during embryogenesis, having a prevalence rate of 0.4 % in the general population [1,2]. When Mullerian ducts fail to fuse during embryogenesis, there is a double uterus with a single cervix and vagina [3]. Congenital uterine malformations like bicornuate uterus increase the risk of infertility, endometriosis, painful hematometra and urinary tract anomalies in non-pregnant women. Whereas in pregnant women, it may increase the risk of spontaneous abortions, preterm deliveries, cervical insufficiency and uterine rupture [1]. A bicornuate uterus is a potential risk factor for uterine rupture [4].

Uterine rupture is an emergency obstetrical condition with high morbidity and mortality and is usually associated with prior cesarean section [5]. Spontaneous uterine rupture usually occurs with ectopic pregnancy, however, its association with a bicornuate uterus is uncommon [6]. The uterine rupture usually occurs in the third trimester or during labor and is very rare during the first or second trimester; thus, diagnosis is challenging and is usually misdiagnosed. Most of the time, an accurate diagnosis is made intraoperatively [7] [8]. Clinical presentations of uterine rupture may be concealed by the changes that occur during pregnancy, thus making the diagnosis more challenging [8]. Lack of a high index of suspicion and poor awareness among healthcare practitioners usually results in delayed diagnosis, which might lead to disastrous outcomes [7].

This report highlights a case of complete, spontaneous rupture of the right rudimentary horn of a bicornuate uterus in a pregnant woman at the second trimester. With the help of this case, we aim to raise awareness about the potential complications of uterine anomalies and the necessity of careful monitoring of pregnancies complicated by such anomalies. This case report has been reported in line with the SCARE checklist [9].

## 2 Case report

A 32-year-old woman (gravida 3, parity 1, living 1, and abortion 1) presented at 20 weeks and 2 days of gestation with a history of amenorrhea for five months, pain in the abdomen for 16 h, and 3 episodes of vomiting. She was afebrile and she didn't complain of vaginal bleeding. She had delivered a child through a caesarian section 8 years ago and had one miscarriage in the past with no other significant past medical and family history.

She was pale and ill-looking on physical examination. Her blood pressure was 110/50 mmHg, and she had tachycardia (pulse rate of 120 beats per minute). The abdomen was distended on examination with the presence of guarding, rigidity, and rebound tenderness. She was initially evaluated in another center and referred to our center for possible perforated appendicitis or ruptured ectopic pregnancy.

An abdominal ultrasound was performed, and it revealed a suspicious defect of approximately 1.5 cm in size in the fundus of the uterus with the presence of mild fluid in the abdomen and pelvis. The clinical suspicion of a ruptured uterus was made.

Laboratory tests showed a hemoglobin level of 5.2 g/dL with normal leucocytes and platelet count. Results of other investigations were not remarkable.

**Fig. 1:**
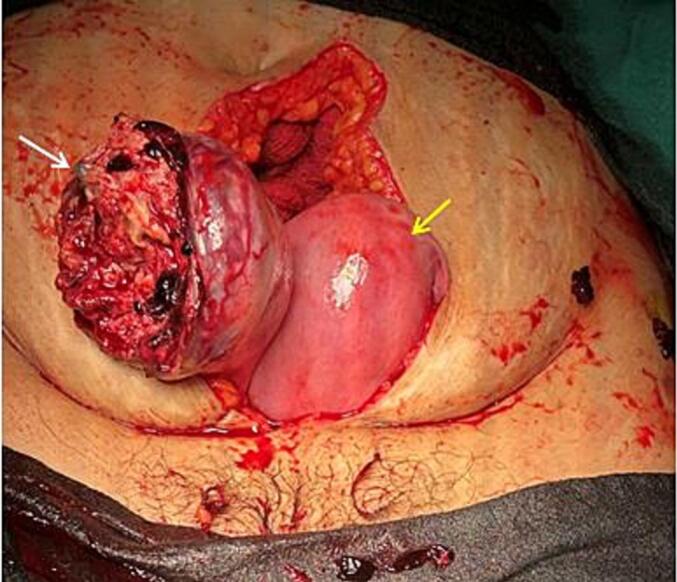
Ruptured right horn of a bicornuate uterus (indicated by white arrow) with normal left horn (indicated by yellow arrow). (For interpretation of the references to colour in this figure legend, the reader is referred to the web version of this article.)

**Fig. 2:**
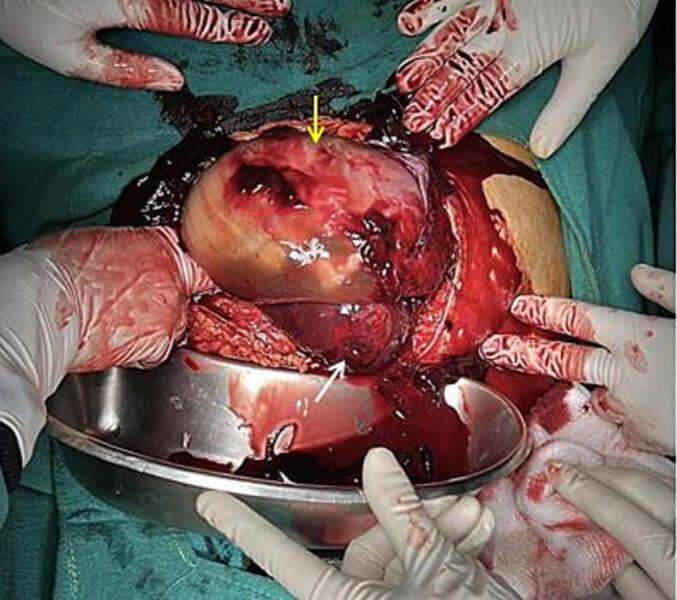
Fetus in the abdominal cavity with intact amniotic membrane (indicated by yellow arrow) with torn edge of the right uterine horn (indicated by white arrow). (For interpretation of the references to colour in this figure legend, the reader is referred to the web version of this article.)

**Fig. 3:**
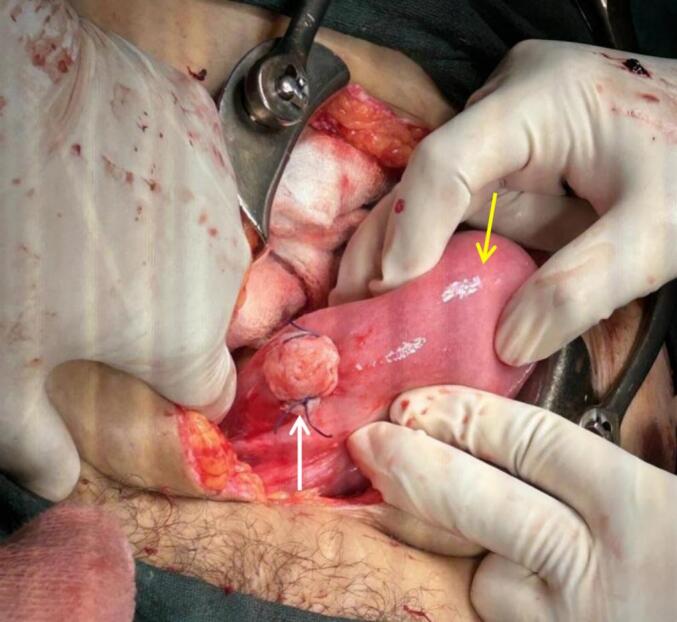
After removal of the right rudimentary horn and product of conception showing stump of the Resected Right Uterine Horn (indicated by white arrow) with intact left horn (indicated by yellow arrow). (For interpretation of the references to colour in this figure legend, the reader is referred to the web version of this article.)

**Fig. 4:**
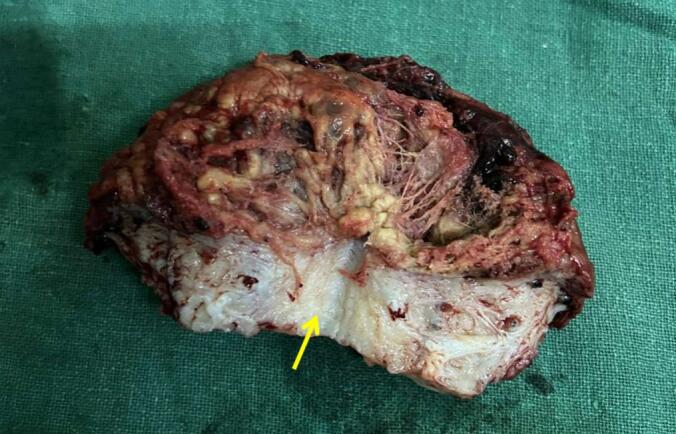
Cut section of excised right horn showing myometrial wall (indicated by an arrow), hemorrhages and clots.

Immediately the patient was taken for exploratory laparotomy with a preoperative diagnosis of ruptured uterus with hemoperitoneum. Three pints (∼1.5 L) of blood were transfused. Intraoperatively, about 1200 mL of blood and clots were obtained on opening the abdomen. The right uterine horn (∼6*5 cm) of the bicornuate uterus was completely ruptured (Fig. 1) with complete extrusion of the fetus in the abdominal cavity with intact amniotic membrane (Fig. 2). Placenta was attached to the uterus, suggestive of placenta increta. The left horn of the uterus was normal with the previous scar of the caesarian section normal and intact. The fetus and placenta were removed. The right round ligament was clamped, cut, and transfixed. The right fallopian tube and ovaries were dissected and freed from the uterus. After an application of clamps at the base of the right rudimentary horn, excision of the right horn was done (Fig. 3). A cut section of the excised right horn is shown in (Fig. 4).

## 3 Discussion

Uterine rupture is an obstetric emergency possessing a high risk of morbidity and mortality [5]. Ectopic pregnancy, uterine anomalies, previous cesarean section, advanced maternal age, grand multiparous, placenta increta, macrosomia, shoulder dystocia, and medical termination of pregnancy are some contributing factors to uterine rupture [10]. Uterine rupture usually occurs in the third trimester or during labor [7], but it can sometimes manifest in the second trimester, especially in the presence of uterine anomalies such as bicornuate uterus [11]. Second-trimester rupture usually occurs at the fundus (as in this case) in contrast to lower uterine segment rupture during labor [12]. This report describes a rare case of second-trimester spontaneous rupture of a bicornuate uterus, leading to hemoperitoneum and intra-uterine fetal loss. Though, our patient had a previous cesarean section but uterine rupture occurred in the right horn while the previous scar was intact on the left horn of the bicornuate uterus making it a rare presentation.

Diagnosis of bicornuate uterus is possible via gynecological sonography, more specifically Sono hysterosalpingography and magnetic resonance imaging. Such procedures are not done routinely on asymptomatic patients especially in developing countries like Nepal because these tests are expensive. Thus, a bicornuate uterus may not be detected until pregnancy or delivery [12]. Most of the women with bicornuate uterus remain asymptomatic and they are diagnosed with this anomaly during pregnancy, childbirth complications, or during hysterectomy [3]. In our case, the bicornuate uterus was diagnosed during explorative laparotomy, indicating failure to its early diagnosis. Although our patient had a previous history of cesarean section, we couldn't find any documentation regarding bicornuate uterus. The patient was not counseled about the condition. Careful evaluation and identification of the bicornuate uterus during a previous cesarean section might have prevented disastrous complications as in our case.

At an early gestation, uterine rupture usually presents with a nonspecific clinical presentation; making its diagnosis more difficult [13]. Clinical presentations include severe acute abdomen, per vaginal bleeding, hypotension, and tachycardia [13]. However, not all cases present these features; thus, it is essential to maintain a high degree of suspicion while managing women who present with such signs and symptoms regardless of the gestational age [13]. In this case, the patient presented with abdominal pain, abdominal distension, guarding and rigidity, vomiting, hypotension, and tachycardia, suggestive of hypovolemia; and abdominal ultrasonography revealed the hemoperitoneum.

The ruptured bicornuate uterus usually necessitates surgical management which includes either resection of the ruptured horn followed by repair as we did in our case or a hysterectomy can be an another option [14]. We managed the case with immediate fluid resuscitation, blood transfusion maintaining hemodynamic stabilization, followed by exploratory laparotomy, and excision of the ruptured right rudimentary horn of the uterus.

## 4 Conclusion

This case highlights the importance of early recognition and prompt management of uterine anomalies like bicornuate uterus to prevent life-threatening complications such as uterine rupture. The ruptured uterus should come as one of the differential diagnoses of acute abdominal pain and features suggestive of hypovolemia and hemoperitoneum in women with second-trimester pregnancies.

## Patient consent

Informed consent was obtained from the patient's family for the publication of this case report, including the use of de-identified imaging and clinical data.

## Ethical approval

Since this is a case report, our Institutional Review Board Institute of Medicine (IOM) has waived the requirement for ethical approval.

## Funding

This research did not receive any specific grant from funding agencies in the public, commercial, or not-for-profit sectors.

## Author contribution

**Sagar Bishowkarma** contributed the conception of the case report, drafting the manuscript, undertaking the literature review and revising the article critically for important intellectual content.

**Sasmit Ghimire** contributed to drafting the manuscript, conception of the case report, and acquiring and interpreting the data.

**Raman Kumar Gurmaita** contributed the conception of the case report, acquiring and interpreting the data.

**Manisha Gurung** contributed to patient care, the conception of the case report, and drafting the manuscript.

**Manvi Mukherjee** contributed to patient care, and revising the article critically for important intellectual content.

**Hema Rijal** contributed to patient care, the conception of the case report, and revising the article for important intellectual content.

## Guarantor

Sasmit Ghimire.

## Research registration number

None.

## Conflict of interest statement

The authors declare that they have no conflict of interest.

## References

[1] Chan Y.Y., Jayaprakasan K., Zamora J., Thornton J.G., Raine-Fenning N., Coomarasamy A. (2011 Nov 1). The prevalence of congenital uterine anomalies in unselected and high-risk populations: a systematic review. Hum. Reprod. Update.

[2] Ludwin A., Pfeifer S.M. (2019 Sep). Reproductive surgery for müllerian anomalies: a review of progress in the last decade. Fertil. Steril..

[3] Jayaprakash S., Muralidhar L., S G., Sexsena R. (2011 Oct 28). Rupture of bicornuate uterus. Case Rep..

[4] Nahum G.G. (2005 Nov). Uterine anomalies, induction of labor, and uterine rupture. Obstet. Gynecol..

[5] Miller D. (1997 May). Intrapartum rupture of the unscarred uterus. Obstet. Gynecol..

[6] Puri M., Jain S., Goyal U. (2000 Aug). Spontaneous rupture of pregnancy in a hemicorpus of complete bicornuate uterus. Aust. N. Z. J. Obstet. Gynaecol..

[7] Ibrahim S., Drymiotou S., Hegab K., Sideris M., Okba M. (2021 Jan). Second trimester rupture uterus, unusual presentation. Ann. Med. Surg..

[8] Arbab F., Boulieu D., Bied V., Payan F., Lornage J., Guerin J.F. (1996 May 1). Uterine rupture in first or second trimester of pregnancy after in-vitro fertilization and embryo transfer. Hum. Reprod..

[9] Kerwan A., Al-Jabir A., Mathew G., Sohrabi C., Rashid R., Franchi T., Nicola M., Agha M., Agha R.A. (2025). Revised surgical CAse REport (SCARE) guideline: an update for the age of artificial intelligence. Prem. J. Sci..

[10] Abdulwahab D., Ismail H., Nusee Z. (2014 Jul 1). Second-trimester uterine rupture: lessons learnt. Malays. J. Med. Sci. MJMS.

[11] Tsegaye M.A., Mekonnen Z.A., Lemma D.T., Adugna A.N., Tesfay R.H. (2025 Mar). Second-trimester uterine rupture in bicornuate uterus: A case report. Case Rep. Womens Health.

[12] Singh N., Singh U., Verma M.L. (2013 Jan). Ruptured bicornuate uterus mimicking ectopic pregnancy: a case report. J. Obstet. Gynaecol. Res..

[13] Saleem H.A., Edweidar Y., Salim M.A., Mahfouz I.A. (2023 Sep). Mid-trimester spontaneous rupture of a bicornuate uterus: a case report. Case Rep. Womens Health.

[14] Heinonen P.K. (2000 Aug). Clinical implications of the didelphic uterus: long-term follow-up of 49 cases. Eur. J. Obstet. Gynecol. Reprod. Biol..

